# Darkening of the Greenland Ice Sheet: Fungal Abundance and Diversity Are Associated With Algal Bloom

**DOI:** 10.3389/fmicb.2019.00557

**Published:** 2019-03-21

**Authors:** Laura Perini, Cene Gostinčar, Alexandre Magno Anesio, Christopher Williamson, Martyn Tranter, Nina Gunde-Cimerman

**Affiliations:** ^1^Department of Biology, Biotechnical Faculty, University of Ljubljana, Ljubljana, Slovenia; ^2^Department of Molecular and Biomedical Sciences, Jožef Stefan Institute, Ljubljana, Slovenia; ^3^Bristol Glaciology Centre, School of Geographical Sciences, University of Bristol, Bristol, United Kingdom; ^4^Department of Environmental Science, Aarhus University, Roskilde, Denmark

**Keywords:** fungi, bacteria, Greenland Ice Sheet, dark ice, ice algae, NGS, microbial diversity, albedo effect

## Abstract

Recent studies have highlighted the importance of ice-algal blooms in driving darkening and therefore surface melt of the Greenland Ice Sheet (GrIS). However, the contribution of fungal and bacterial communities to this microbially driven albedo reduction remains unconstrained. To address this significant knowledge gap, fungi were isolated from key GrIS surface habitats (surface ice containing varying abundance of ice algae, supraglacial water, cryoconite holes, and snow), and a combination of cultivation and sequencing methods utilized to characterize the algal-associated fungal and bacterial diversity and abundance. Six hundred and ninety-seven taxa of fungi were obtained by amplicon sequencing and more than 200 fungal cultures belonging to 46 different species were isolated through cultivation approaches. Basidiomycota dominated in surface ice and water samples, and Ascomycota in snow samples. Amplicon sequencing revealed that bacteria were characterized by a higher diversity (883 taxa detected). Results from cultivation as well as ergosterol analyses suggested that surface ice dominated by ice algae and cryoconite holes supported the highest fungal biomass (10^4^–10^5^ CFU/100 ml) and that many fungal taxa recognized as endophytes and plant pathogens were associated with dark ice characterized by a high abundance of ice algae. This paper significantly advances this field of research by investigating for the first time the fungal abundance and diversity associated with algal blooms causing the darkening of the GrIS. There is a strong association between the abundance and diversity of fungal species and the blooming of algae on the surface ice of the Greenland Ice Sheet.

## Introduction

The Greenland Ice Sheet (GrIS) is the largest ice mass of the northern hemisphere, covering an area of around 1.7 million km^2^ and comprising circa 11% of the Earth’s cryosphere (Abdalati and Steffen, 1995). Recently recognized as one of the Earth’s biomes (Anesio and Laybourn-Parry, 2012), the GrIS is dominated by various microbial communities that inhabit a range of surface environments, including snow, ice, supraglacial water and cryoconite holes formed via a bio-cryomorphological process (Cook et al., 2015). Significant research is currently focused on ice algal taxa belonging to the class Zygnematophyceae (Streptophyta) that are able to survive in surface ice environments (Remias et al., 2009, 2012a,b, Williamson et al., 2018). These algae are the closest living relatives of land plants (de Vries et al., 2016; de Vries and Archibald, 2018) and can form extensive blooms within surface ice during summer ablation seasons (Yallop et al., 2012; Stibal et al., 2017; Williamson et al., 2018). Such algal blooms are typically dominated by *Ancylonema nordenskiöldii* and *Mesotaenium berggrenii* (Williamson et al., 2018) and, due to the high abundance of cells apparent (∼10^4^ cells ml^-1^) and the significant pigmentation of ice algal taxa (Remias et al., 2009, 2012a,b, Williamson et al., 2018), impart a conspicuous brownish-grayish tint to the supraglacial ice, which is subsequently referred to as dark or dirty ice (Williamson et al., 2018). Together with wind-borne debris, ice algal blooms thus serve to reduce GrIS surface reflectance (albedo) (Yallop et al., 2012; Musilova et al., 2017; Stibal et al., 2017; Ryan et al., 2018) and consequently, increase surface melt (van den Broeke et al., 2017; Ryan et al., 2018). Increased surface melt in turn supplies more melt water to the microorganisms, creating a positive feedback loop between their growth and melting of the GrIS.

To-date, the majority of microbial studies conducted on GrIS supraglacial habitats have focused on ice algal blooms (Yallop et al., 2012; Stibal et al., 2017; Williamson et al., 2018), and the abundance and diversity of bacterial communities on ice and cryoconites (Cameron et al., 2015a,b; Musilova et al., 2015; Stibal et al., 2015). However, to our knowledge, no studies have addressed the diversity or abundance of GrIS fungal communities, or their potential associations with the ice algal community. Fungi are otherwise recognized as an important part of the Arctic’s microbial biodiversity, with over 4,000 species described to-date (Dahlberg et al., 2013). Fungal communities are known to be essential for the functioning of Arctic ecosystems given their saprotrophic roles (Dahlberg et al., 2013), and in bio-geochemical cycling processes (Gadd, 2007). Fungi also influence the occurrence of other microorganisms through different types of interactions, beneficial as well as detrimental (Meltofte et al., 2013; Rämä et al., 2017). However, the fungal impact on the biogeochemical processes within different GrIS habitats remain underestimated. In Greenland, investigations of fungi have been limited to the studies of their occurrence, distribution and ecology in soil and in association with lichens (Alstrup et al., 2000; Zoller and Lutzoni, 2003; Kristinsson et al., 2010; Timling and Taylor, 2012; Timling et al., 2014), with no data available on fungi proliferating and interacting in water-ice-based environments.

The aim of this study was therefore to provide the first comprehensive analysis of the fungal diversity from the surface of the GrIS. To this end, a combination of culturing and ITS amplicon sequencing was applied to five different GrIS surface habitats: snow, supraglacial water, sediment and water from cryoconite holes, and surface ice containing a low and high abundance of ice algae. Bacterial diversity was determined in parallel by cultivation and amplicon sequencing techniques. Special consideration was given to the comparison between the dark ice and other habitats.

## Materials and Methods

### Site and Sampling Description

**FIGURE 1:**
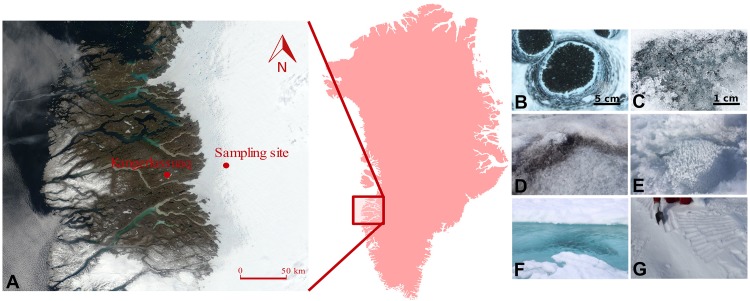
Location of the sampling site on the south-western Greenland Ice Sheet **(A)**, with insert showing the relative position of the sampling region within Greenland. Map is acquired as an image from Modis Satellite. Images show the type of samples collected: cryoconite holes **(B)**, dispersed cryoconite **(C)**, dark ice **(D)**, clear ice **(E)**, supraglacial water **(F)**, and fresh snow **(G)**.

Two sampling campaigns were conducted during the 2016 (July–August) and 2017 (June–July) melt seasons in the south-western ablation zone of the GrIS, covering in 2 years an entire ablation season: from snow to the end of melt. The sampling site was located ∼60 km (67.078694, -49.341583) east of Kangerlussuaq, and was situated within the ‘dark zone,’ a region running along the western edge of the GrIS that is characterized by particularly low albedo and extensive ice algal blooms during summer melt seasons (Yallop et al., 2012; Stibal et al., 2017; Williamson et al., 2018) (Figure 1). The samples were collected using clean, disposable nitrile gloves, and were transferred into sterile *Whirl-Pak*^®^ plastic bags. Samples were collected from supraglacial water, sediment and water from cryoconite holes (“slurry”), dispersed cryoconite (only in 2016), fresh snow (only in 2017), surface ice with high abundance of ice algae (10^4^ cells ml^-1^) – hereafter referred to as dark ice, and with low algal abundance (10^1^ cells ml^-1^), referred to as clear ice.

### Cultivation-Based Fungal and Bacterial Diversity Analysis

Initial sampling to investigate the presence/absence of surface fungal communities was performed in 2016 of which cultivation and NGS results will be shown, with more comprehensive sampling and analyses performed in 2017 given our initial results. All samples, except supraglacial water and slurry from cryoconite holes, were melted aseptically *in situ* at site temperature and due to the unknown fungal abundances and in order to obtain single colony forming units, aliquots of 10 ml and 100 ml were filtered through Milli-pore membrane filters (0.45 μm pore size) in duplicate. Filters were placed on two enumeration and four different selective agar media with either low nutrient content (used only in 2017 sampling) or with low water activity (a_w_). 100 μl of the original sample from all environments were directly applied on all media and dispersed with a sterile Drigalski spatula. Media used included DRBC – a general-purpose enumeration medium (a_w_ = 1) (King et al., 1979); DG-18 – a medium for detection of moderate xerophiles (a_w_ = 0.946) (Hocking and Pittx, 1980); MY10-12 – a medium for the isolation of xero- and halo-tolerant fungi with 10% glucose and 12% NaCl (a_w_ = 0.880) (Pitt and Hocking, 2009); R2A – a low nutrient enumeration medium for heterotrophic microorganisms (both bacteria and fungi), (a_w_ = 1), (Reasoner and Geldreichm, 1985); and SNA and MM – two nutrient-poor media for the isolation of oligotrophic fungi (a_w_ = 1) used in 2017 sampling (Nirenberg, 1981; de Vries et al., 2004). For the prevention of bacterial growth, chloramphenicol (50 mg/l) was added to all media, except for R2A agar. Plates, with a total of seven plates of each medium for each sample, were incubated at the sampling site at temperatures varying from approximately -2°C to +10°C for 1 week, and at 10°C for 4–12 weeks at the University of Ljubljana, where subsequent analyses were carried out. For every medium, the average number of colony forming units (CFU/100 ml) was visually determined.

### Fungal and Bacterial Identification

DNA was extracted from pure fungal and bacterial cultures up to 1 week after incubation on a malt extract agar (MEA) and R2A media, respectively. DNA from filamentous fungi was extracted by mechanical lysis of approximately cm^2^ of mycelium according to van den Ende and de Hoog (1999). For yeast-like and bacterial strains, DNA was extracted using PrepMan Ultra reagent (Applied Biosystems, Foster City, CA, United States) according to the manufacturer instructions. For filamentous fungi a fragment of rDNA including ITS region 1, 5.8S rDNA and ITS region 2 (ITS) was amplified using ITS5 and ITS4 primers (White et al., 1990). Polymerase chain reactions (PCRs) were performed using Thermo Scientific *Taq* DNA Polymerase according to manufacturer’s protocol. Reactions were run in a PCR Mastercycler Ep Gradient (Eppendorf) with an initial denaturation of 2 min at 95°C, followed by 30 cycles of denaturation at 95°C for 45 s, annealing at 54°C for 30 s, and elongation at 72°C for 2 min, with a final elongation at 72°C for 4 min. For identification of *Penicillium* strains, the partial β-tubulin gene (benA) was amplified and sequenced with Ben2f and Bt2b primers (Glass and Donaldson, 1995). Initial denaturation at 95°C for 1 min was followed by 35 cycles of denaturation at 95°C for 30 s, annealing at 53°C for 30 s, and elongation at 72°C for 1 min. Final elongation was at 72°C for 10 min. *Cladosporium* strains were identified using partial actin (act) sequences, amplified with ACT-512F and ACT-783R primers (Carbone and Kohn, 1999). Initial denaturation at 94°C for 5 min was followed by 45 cycles of denaturation at 94°C for 45 s, annealing at 52°C for 30 s and elongation at 72°C for 90 s. Final elongation was at 72°C for 5 min. For yeasts domains D1 and D2 of LSU rDNA gene were amplified using NL1 and NL4 primers (Boekhout and Kurtzman, 1996). Initial denaturation at 95°C for 5 min was followed by 30 cycles of denaturation at 95°C for 45 s, annealing at 54°C for 30 s and elongation at 72°C for 2 min. Final elongation was at 72°C for 4 min. For bacteria 16S rRNA gene was amplified with 27f-lane and 1492R primers (Lane, 1991) and a touchdown program. Initial denaturation at 95°C for 5 min was followed by five cycles of denaturation at 95°C for 30 s, annealing at 60°C for 30 s and elongation at 72°C for 1 min; five cycles of denaturation at 95°C for 30 s, annealing at 55°C for 30 s and elongation at 72°C for 1 min; and 30 cycles of denaturation at 95°C for 30 s, annealing at 50°C for 30 s and elongation at 72°C for 1 min. Final elongation was at 72°C for 7 min. The ITS, LSU, benA, act and 16S nucleotide sequences were determined by Sanger sequencing, performed by Microsynth AG, Switzerland. The resulting sequences of all the isolates were aligned using MUSCLE software (Edgar, 2004) implemented in MEGA5 package (Tamura et al., 2011) and examined using the BLAST software of the National Centre for Biotechnology Information (NCBI) database (Altschul et al., 1990). Maximum likelihood methods implemented in PhyML 3.0 (Guindon et al., 2010) were used to build phylogenetic trees aligning the resulting sequencing with type and reference sequences in MEGA5 (Tamura et al., 2011) (Supplementary Material). All isolated strains have been deposited in the Ex F Culture Collection of the Infrastructural Centre Mycosmo (MRIC UL) at the Department of Biology, Biotechnical Faculty, University of Ljubljana, Slovenia. LSU, ITS, and 16S sequences have been deposited in the GenBank database. GenBank accession numbers are available in the Supplementary Material.

### Fungal and Bacterial Diversity Analysis Using Illumina Sequencing

For 2016 samples, DNA was isolated from filtered biomass (sample volume 750 ml, 0.45 μm pore size, Millipore) of clear ice (C-ice), dark ice (D-ice) and supraglacial water (S-wtr). From 2017 samples, DNA was isolated from 700 ml of dark ice, 1 L of clear ice, 2 L of melted snow (Snow), and 1 L of supraglacial water. With the exception of supraglacial water from 2016, two biological replicates of each sample were assessed. All filters and 1 g of cryoconite sediment (Cry) were placed in 1.5 ml microcentrifuge tubes containing RNAlater^®^ (Sigma-Aldrich Company Ltd., United Kingdom) and immediately frozen at -20°C until further analysis in the laboratory. Replicates were treated as independent, with a total of 15 samples labeled with corresponding habitat type followed by the collection year and the number of replicate: C-ice16-1; C-ice16-2; C-ice17-1; C-ice17-2; Cry17-1; Cry17-2; D-ice16-1; D-ice16-2; D-ice17-1; D-ice17-2; S-wtr16-1; S-wtr17-1; S-wtr17-2; Snow17-1; Snow17-2. DNA was extracted from filters and cryoconite sediment using the PowerWater DNA Isolation Kit (MoBio Laboratories Inc., Carlsbad, CA, United States) and then from the same filters using the PowerLyzer PowerSoil DNA Isolation Kit (MoBio Laboratories Inc., Carlsbad, CA, United States), according to the manufacturer’s instructions with a slight modification to increase the DNA yield and quality. To increase efficiency of fungal cells lysis an additional heating incubation at 65°C for 10 min was used after adding PW1 solution. DNA from both isolation methods was pooled together and stored at -80°C until PCRs were performed.

For the analysis of fungal diversity, Illumina Miseq V3 (300 bp paired-end) sequencing was carried out on the ITS2 region of the ITS rDNA gene amplified using the primers ITS4-Fun (5′ AGCCTCCGCTTATTGATATGCTTAART-3′) and 5.8S-Fun (5′-AACTTTYRRCAAYGGATCWCT-3′) (Taylor et al., 2016). Amplification was carried out in a PCR Mastercycler Ep Gradient (Eppendorf) with initial denaturation of 2 min at 98°C, followed by 20 cycles of 10 s at 98°C, 25 s at 54°C and of 25 s at 72°C, with a final elongation of 7 min at 72°C.

For analysis of bacterial diversity, Illumina Miseq V2 (250 bp paired-end) sequencing was carried out on the hypervariable V3 and V4 regions of the 16S rRNA gene by using the 341F_ill (5′-CCTACGGGNGGCWGCAG-3′) and 802R_ill (5′-GACTACHVGGGTATCTAATCC-3′) universal bacterial primers (Klindworth et al., 2013). Due to the low amount of DNA, the first step PCRs were performed in Ljubljana lab using Phusion^®^ High-Fidelity DNA Polymerase according to manufacturer’s protocol. Reactions were run in a PCR Mastercycler Ep Gradient (Eppendorf) with initial denaturation of 3 min at 95°C, followed by 20 cycles of 20 s at 95°C, 30 s at 56°C and of 30 s at 72°C, with a final elongation of 5 min at 72°C.

Single-end reads were quality checked and trimmed (minimum quality score 20) and analyzed with QIIME2 2018.8 software package (Quantitative Insights Into Microbial Ecology) (Caporaso et al., 2010). The forward reads data were denoised by DADA2 (Callahan et al., 2016) algorithm using the default parameters, the tree was constructed by FastTree on a mafft alignment and rooted at midpoint and the alpha and beta diversity indices were calculated. For assigning the taxonomy to sequences, the 99% cut-off GreenGene database (McDonald et al., 2012) was used for training the feature classifier for bacteria, and the dynamically clustered UNITE ITS database (Abarenkov et al., 2010) was used for fungi. Abundances in each sample were normalized to the number of sequences in the least abundant sample. Due to the high presence of ice algae in dark and clear ice samples, and in order to investigate the bacterial diversity in all samples in a comparable manner, chloroplast sequences were excluded using a taxonomy-based filtering. Shannon index was used to study the alpha diversity. The distance and dissimilarity matrix were determined through Bray–Curtis index to visualize the ordination and clustering of the bacterial and fungal community composition for beta diversity analyses using the principal coordinate analysis (PCoA). Differences in microbial community composition between sample types were assessed by non-parametric permutational analysis of variance (PERMANOVA). All analyses above were performed in QIIME2 2018.8 software package (Quantitative Insights Into Microbial Ecology) (Caporaso et al., 2010).

### Ergosterol Quantification

Quantification of ergosterol as a biomarker molecule for the determination of fungal presence was performed for 2017 environmental samples. Filtration of 500 ml of melted snow and 100 ml of supraglacial water, dark ice, clear ice and cryoconite was performed through Milli-pore membrane filters (0.45 μm pore size) in biological triplicates (in duplicate for snow). Filters were then stored in sterile microcentrifuge tubes containing 1.5 ml of 90% methanol and frozen *in situ* until analysis. In the laboratory (Ljubljana), filters and corresponding methanol were transferred to 5 ml sterile centrifuge tubes, and 3 ml of 100% chloroform was added and vortexed for 15 min at room temperature. The methanol phase was transferred to a sterile 1.5 ml microcentrifuge tube and dried in a biosafety cabinet overnight. The residues were re-dissolved in 1.5 ml 90% methanol and vortexed for 5 min at room temperature. Samples were then filtered through 0.22 μm pore size filters and transferred to HPLC tubes. HPLC analyses were performed using Waters HPLC system 2965 (Milford, MA, United States), equipped with a degasser, a quaternary pump, an auto-sampler and 2998 PDA detector set at 280 nm. A Kinetex XB-C18 (150 × 4.6 mm, particle size 5 μm) column (Phenomenex, Torrance, CA, United States) was used. 50 μl of each sample was injected. Ergosterol was eluted from the column using an isocratic mobile phase of 100% methanol and a flow rate of 1.0 ml/min. Before HPLC analysis, standard ergosterol (Sigma, St. Louis, MO, United States) was purified according to Nylund and Wallander (1992). Ergosterol peak was identified by comparison of the retention time and absorbance spectra with the purified ergosterol as standard. Correlation analyses between fungal abundance and ergosterol quantification were performed in R (R Core Team, 2017).

## Results

### Quantification of Cultivable Fungi

**FIGURE 2:**
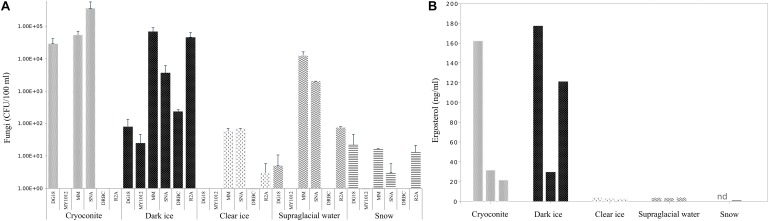
Fungal abundances **(A)** in CFU/100 ml and ergosterol concentration **(B)** in ng/ml in cryoconite, dark ice, clear ice, supraglacial water and snow. Abundance data were log-transformed and errors bar denoted standard deviation.

Abundances of cultivable fungi from samples collected in 2017 are presented in Figure 2A. Fungal abundances differed considerably depending on the media used. Overall the lowest counts were obtained on low a_w_ media: on MY10-12 up to 2.5 × 10 CFU/100 ml and on DG-18 up to 3 × 10^5^ CFU/100 ml. DRBC counts ranged from 0 to 2.3 × 10^2^ CFU/100 ml. Significantly higher counts were obtained on low nutrient media: on MM up to 7 × 10^4^ CFU/100 ml and on SNA up to 3.6 × 10^5^ CFU/100 ml.

The overall lowest counts were detected in fresh snow, with fungal counts, ranging from 0 (DRBC) to 2 × 10 CFU/100 ml (MM and DG-18). In contrast, samples from cryoconites holes had the highest fungal abundance with counts ranging from 5 × 10^4^ CFU/100 ml (DG-18 and MM) to 3.6 × 10^5^ CFU/100 ml on SNA. Fungal abundance in dark ice spanned 2–8 × 10 CFU/100 ml on the two low a_w_ media (MY10-12 and DG-18), but increased considerably on media with low nutrient concentrations (SNA, MM), where CFU ranged from 2 to 7 × 10^4^ CFU/100 ml. Clear ice contained considerably lower counts than cryoconite and dark ice, ranging from 0 on DG-18 and MY10-12 to 6 × 10 CFU/100 ml on MM and SNA. CFU in supraglacial water were considerably higher relative to clear ice and snow and ranged from no isolates (DRBC and MY10-12) to 1 × 10^4^ CFU/100 ml.

### Relative Quantification of Fungal Biomass With Ergosterol

Quantification of ergosterol in samples collected during 2017 fieldwork is shown in Figure 2B.

Ergosterol levels were highest in cryoconite slur and dark ice compared to the other sampled habitats, with concentrations up to 162 ng/ml and 177 ng/ml, respectively. Interestingly, biological replicates of samples with the highest levels showed high variability in ergosterol concentrations, with values ranging between 21.6 and 162 ng/ml in cryoconite slur, and 30 and 177 ng/ml in dark ice. However, even the smallest ergosterol quantities in the abovementioned samples were 10 times higher than any of the samples of fresh snow, clear ice and supraglacial water. Concentrations of ergosterol in supraglacial water and clear ice were similar, with values from 3.6 to 3.9 ng/ml, and 2.4 to 3.6 ng/ml, respectively. The lowest levels of ergosterol, from undetectable to 0.66 ng/ml, were found in fresh snow.

### Cultivable Fungal Diversity

A total of 260 fungal isolates were obtained from all five habitats sampled in 2017. Cultivation identified dark ice as the habitat with the most diverse fungal community, with many of these species plant pathogens or endophytes. Commonly isolated yeasts associated with dark ice were the ascomycetous black yeast *Oleoguttula mirabilis* and two basidiomycetous yeasts, *Glaciozyma antarctica*-like and *Rhodotorula “svalbardensis”.* Other yeasts that were isolated sporadically were *Vishniacozyma victoriae*, *Dothiora* sp., *Mrakia* sp., *Phenoliferia glacialis*, *Sporobolomyces ruberrimus*, *Tilletiopsis washingtonensis* and Basidiomycota sp. 2, 4, 6 (Uetake et al., 2012) (total CFUs in 100 μl of dark ice belonging to yeasts ranged from 2 to 90). Filamentous fungi commonly isolated from dark ice were *Cladosporium* sp., and *Claussenomyces* sp. Less frequently isolated fungi were *Acrodontium luzulae*, *Epicoccum sp.*, *Aspergillus* sp., *Athelia* sp., *Bjerkandera adusta*, *Cadophora* sp., *Comoclathris lini*, *Comoclathris* sp., unidentified *Helotiales*, *Microdochium nivale*, *Penicillium bialowiezense*, and *Venturia* sp. (total CFUs in 100 μl of dark ice belonging to filamentous fungi ranged from 1 to 13). In comparison to dark ice, a much lower recovery of cultivable species was obtained from clear ice. However, *Oleoguttula mirabilis*, *Glaciozyma antarctica*-like and *Rhodotorula “svalbardensis”* remained the most common taxa, with *Baeospora myosura*, Basidiomycota sp. 2 (Uetake et al., 2012), *Coleophoma* sp., and *Penicillium solitum* isolated sporadically. Species characteristic of supraglacial water included basidiomycetous yeast such as *Glaciozyma antarctica*-like, one of the dominant yeasts both in dirty ice and clear ice, and additionally *Ph. glacialis*, *Ph.* sp., Basidiomycota sp. 2 (Uetake et al., 2012), *Glaciozyma watsonii*, and *Mrakia* sp. Filamentous fungi such as *Cladosporium* sp. and unidentified Helotiales were occasionally present.

Despite the low fungal abundance, fresh snow had the second highest fungal diversity – and no dominant species - assessed both by cultivation and molecular methods (see below). Filamentous fungi were represented by *Preussia* sp., *Neocucurbitaria* sp., *Cladosporium* sp., *Comoclathris* sp., *Sydowia polyspora*, *Hyalodendriella betulae*, *Coniochaeta rosae*, *Penicillium chrysogenum*, *P. crustosum*, *P. fusisporum*, *Penicillium* sp., *Thelebolus globosus*, *Venturia* sp., and unidentified Ascomycota. Yeast species included *Aureobasidium pullulans*, *Vishniacozyma carnescens*, unidentified *Dothideomycetes*, *Vishniacozyma victoriae*, *Cystofilobasidium capitatum*, *Dothiora* sp., *Mrakia* sp., and *Tilletiopsis washingtonensis*. The principal component analyses (PCA) of the cultivable fungal diversity showed dark ice and snow as the habitats with the most diverse fungal communities compared to cryoconite, supraglacial water and clear ice (Supplementary Material). Furthermore, SIMPER analyses identified dark ice and snow as the habitats that contributed the most to the overall dissimilarity (Supplementary Material), with some species such as *Acrodontium* *luzulae*, *Epicoccum* sp., *Aspergillus* sp., *Athelia* sp., *Bjerkandera adusta*, *Cadophora* sp., *Claussenomyces* sp., *Microdochium nivale*, *Penicillium bialowiezense*, *Sporobolomyces ruberrimus* present just in dark ice samples.

Cryoconite holes differed from all other communities by a high presence of *Articulospora* sp., present to a lesser extent also in plated ice algae. As in clear and dark ice, *Rhodotorula “svalbardensis”* was the dominant species also in cryoconites. Other sporadic fungal species were *Cladosporium* sp., *Vishniacozyma victoriae*, *Dothiora europaea*, *Mrakia* sp., *Glaciozyma antarctica*-like, and *Preussia* sp.

### Fungal Diversity (Amplicon Sequencing)

In total 4,357,151 single-end reads were obtained from 15 samples, corresponding to 697 different operational taxonomic units (OTUs). The number of reads per sample ranged from 109,100 (Cry17-1) to 434,630 (S-wtr17-1), with the exception of sample S-wtr16, where the number of reads was much lower (39,737), therefore this sample was excluded from analyses of alpha and beta diversity. Sequence data generated and analyzed during the current study are available in GenBank under the BioProject with the accession code PRJNA507743.

Shannon indices (Supplementary Material) varied over a broad range from H′ = 2.28 (D-ice17-1) to H′ = 4.7 (Snow17-2), revealing differences in diversity between habitats. To test for differences between sample types, PERMANOVA multivariate diversity analyses were performed. Significant differences were found between all five types of samples (i.e., dark ice, clear ice, snow, cryoconite hole, supraglacial water, pseudo-F = 2.78; *p* = 0.001; the number of permutations = 999). However, no statistically significant differences were found in the pairwise PERMANOVA comparisons of sample types, possibly due to the small numbers of samples of each sample type.

**FIGURE 3:**
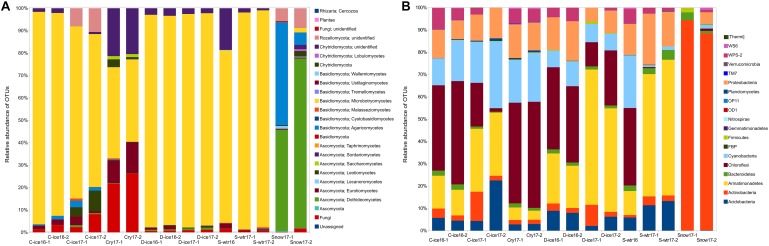
Percent of fungal **(A)** and bacterial **(B)** operational taxonomic units (OTUs) aligned and assigned to known fungal classes/bacterial phyla based on PCR amplifications of ITS2/16S gene sequences for all the sample types.

Clear ice, dark ice and supraglacial water samples from both years (2016 and 2017) were dominated by *Microbotryomycetes* (Basidiomycota) (Figure 3A), with relative frequencies above 68% (frequency of this group was 68.2–94.7% in clear ice, 93.3–95.3% in dark ice, and 76.8–96.8% in supraglacial water). Analyses at lower taxonomic levels revealed that a large part of the sequences within the *Microbotryomycetes* class was unassigned, while 17.2–69.4% were identified to the order Leucosporidiales. Cryoconite samples were still dominated by *Microbotryomycetes* class (36.8–40.7%), followed by Chytridiomycota phylum (22.8–26.3%). The phylum Chytridiomycota was also present in all the other samples, except for snow, with lower frequencies (0.01–2.1% in clear ice, 2.2–3.4% in dark ice, and 0.9–18.6% in supraglacial water). Snow samples were dominated by 45.2–75.6% Dothideomycetes (Ascomycota) and by 45.7–5.5% Agaricomycetes (Basidiomycota). Although black yeast genus *Aureobasidium* (Dothideales) represented 0.7–3% of the Dothideomycetes, the majority of this class (40.5–66.7%) belonged to the Pleosporales, with the two most abundant families being Pleosporaceae (16.2–32.6%) and Sporormiaceae (14.8–23.7%) and the most abundant genus *Comoclathris* (9.4–31.4%). Snow samples and clear ice 2017 samples were characterized by the presence of Rozellomycota phylum (8.6–5.8% and 8–10.6%, respectively). Dark ice from 2016 and 2017, clear ice from 2017 and snow contained *Oleoguttula mirabilis* sequences with a relative abundance <1%. The class Malasseziomycetes was present in clear ice, supraglacial water and cryoconite samples with a frequency <1%.

**FIGURE 4:**
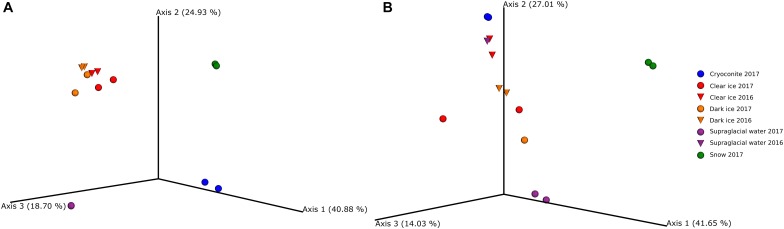
Principal coordinate analysis (PCoA) ordination patterns based on Bray–Curtis dissimilarity index of fungi **(A)** and bacteria **(B)**.

The PCA of ordination patterns revealed four separate sample clusters based on Bray-Curtis dissimilarity index (Figure 4A). The three axes of PCoA accounted for 84.5% of the variation. Dark ice samples clustered together with clear ice samples, while cryoconite, supraglacial water, and snow samples formed separate clusters, reflecting the distinct microbial communities of these samples types.

### Quantification of Cultivable Bacteria (R2A Medium)

**FIGURE 5:**
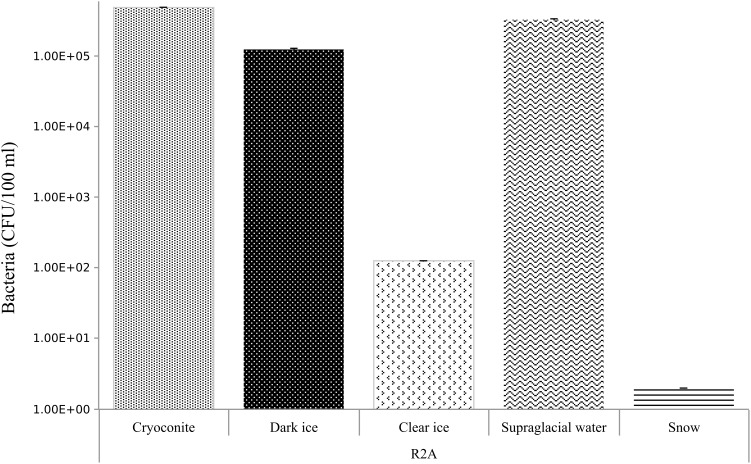
Bacterial abundances in CFU/100 ml in cryoconite, dark ice, clear ice, supraglacial water and snow. Errors bar denoted standard deviation. Abundance data were log-transformed.

Abundances of bacteria on R2A medium are summarized in Figure 5. Cryoconite, dark ice and supraglacial water were the samples with the highest bacterial counts, from 5.0, 1.0, 3.0 × 10^5^ CFU/100 ml, respectively. Clear ice followed with 1.3 × 10^2^ CFU/100 ml, while fresh snow had the lowest bacterial counts – 2 CFU/100 ml.

### Diversity of Cultivable Bacteria

Forty-six bacterial isolates were obtained from all five environments sampled in 2017. Cryoconites had the most diverse bacterial community. The most isolated species was *Pseudomonas* sp., followed by *Rugamonas rubra*, *Janthinobacterium* sp. and *Undibacterium* sp. Amongst sporadically isolated species were *unidentified Oxalobacteraceae*, and *Sphingomonas* sp.

In dark ice isolates belonged to *Bacillus* sp., *Curtobacterium* sp., *Frigoribacterium* sp., uncultured Oxalobacteraceae similar to isolates from Arctic sea ice (Brinkmeyer et al., 2003), *Rugamonas rubra*, and *Sphingomonas glacialis*. In clear ice the majority of isolates were *Cryobacterium* sp., but also species such as *Rugamonas rubra*, *Bacillus* sp., *Frigoribacterium* sp., and an unidentified species belonging to Oxalobacteraceae family were found.

Supraglacial water was dominated by so far uncultured species belonging to Oxalobacteraceae family, *Janthinobacterium* sp., and *Massilia* sp. *Mesorhizobium* sp. and *Sphingomonas* sp. were sporadic. In snow samples the only isolate belonged to *Curtobacterium* sp.

### Diversity of Bacteria (Amplicon Sequencing)

After subtraction of chloroplast sequences, the dataset was composed of 605,531 assembled sequences (for 15 samples in total), corresponding to 883 different features OTUs. Number of reads per sample ranged from 2,067 (D-ice17-1) to 108,084 (S-wtr17-1). Sequence data generated and analyzed during the current study are available in GenBank under the BioProject with the accession code PRJNA517071.

Shannon indices (Supplementary Material) for bacteria varied over a broad range from H′ = 2.73 (Snow17-1) to H′ = 5.74 (S-wtr16). Samples with lower bacterial diversity indices were C-ice17-2, D-ice17-1 and 2, Snow17-1 and 2. PERMANOVA multivariate diversity showed differences between all five sample types (i.e., dark ice, clear ice, snow, cryoconite, supraglacial water, pseudo-F = 2.38; *p* = 0.001, and the number of permutations = 999). Pairwise PERMANOVA identified statistically significant differences between dark ice and supraglacial water (*p* < 0.005), but no other significant differences were found (as in the case of fungi, possibly due to a small number of samples per sample type).

The common bacterial phyla across all 15 samples were Actinobacteria, Bacteroidetes, Proteobacteria, and Cyanobacteria, with broad ranges of relative abundances (1.1–94.4%, 0.4–4.4%, 0.3–22.8%, 0.8–29.9%, respectively) (Figure 3B). However, many taxa were environment-specific. For instance, snow was dominated by the genus *Micrococcus* (Actinobacteria; 86.5–94.4%), which was represented by few sequences in other samples (<1%), while the genus *Hymenobacter* (Bacteroidetes) and the phylum Acidobacteria were present in all other samples except snow. Phylum Armatimonadetes was present in ice and supraglacial water from 2017 in high abundance (10.8–60.8%). Its presence was lower in cryoconites (4.17–5.6%), while it was absent from snow. Cryoconite samples were characterized by the classes Chloroflexi (mainly class Ktedonobacteria) and WPS-2. Genus *Polaromonas* was found in ice from 2016 and supraglacial water from both sampling years.

The PCA of ordination patterns revealed five separate sample clusters based on Bray–Curtis dissimilarity index (Figure 4B). The three axes of the PCoA accounted for 82.7% of the variation. Samples of clear ice, and one replicate of dark ice from 2017 clustered together. The second cluster was composed of supraglacial water and dark ice samples, both from 2016, and cryoconite. As in the case of fungal diversity, snow and supraglacial water 2017 were clearly separated from the rest, reflecting a distinct microbial diversity.

## Discussion

This is the first study to characterize the fungal diversity and abundance across different habitats of the surface of the GrIS using both cultivation and sequencing approaches. Particular emphasis was given to algal dominated ice in comparison to other habitats due to its impact on surface albedo and potential to exacerbate surface melt. In this study, we use the information about fungal diversity in the different supraglacial habitats to speculate potential roles of supraglacial fungal community in association with ice algae, such as a saprotrophic role in degradation of the algal biomass, but also possibly pathogenic or symbiotic relationship, either as endophytes relieving stress conditions or akin to those of lichens helping the ice algae in stabilizing themselves on the ice. Interactions between fungi and ice algae may thus influence GrIS ice algal bloom dynamics, and consequently hold potential to indirectly impact ice surface albedo and melt.

### Ice With Blooming Algae Supports a Higher Fungal and Bacterial Abundance

Fungal abundance was estimated across five different surface habitats of the GrIS via cultivation and ergosterol measurements. The relative amounts of ergosterol confirmed the fungal abundance determined by cultivation in cryoconite and dark ice. MM, due to its low nutrient concentration, limits the growth of highly sporulating fungi and thus reduces the overestimation of their number. This may be the explanation of why in our study, MM is the medium that better reflected the real abundance of fungi and that correlated with the ergosterol quantification with a correlation coefficient of 0.726 (*p* = 0.011). Ergosterol on Canadian soil samples measured with HPLC procedure by Montgomery et al. (2000) showed quantifications ranging from 130 to 1,210 ng/ml. Lowest concentration reported in soil is comparable with concentrations measured for cryoconite and dark ice habitats in this study. Although ergosterol is commonly used as biomarker in various types of environmental samples to estimate the abundance of living fungal biomass (Gessner, 2005), this is the first use of ergosterol as a biomarker in glacial ice of Arctic or Antarctic environments. Since ergosterol content can vary considerably between fungal species, growth phase and culturing conditions, converting ergosterol measurements to biomass can be inaccurate. Also, early lineages fungi detected with amplicon sequencing and observed in abundance in some habitats, such as Chytridiomycota, do not synthetize ergosterol (Newell, 2001). On the other hand, the presence of non-fungal ergosterol producers, such as green algae belonging to the genus Chlamydomonas (Brumfield et al., 2017) in the studied habitats is possible. Conversion factors can lead to both an under- or over-estimation of the fungal biomass (Gessner and Newell, 2002); therefore conversion to biomass was not attempted in this study, with data interpreted in a relative manner between habitats.

In contrast to fungi, bacterial abundance in different cold environments (Foreman et al., 2007; Anesio and Laybourn-Parry, 2012) and in Greenland has been determined previously (Stibal et al., 2015). However, in these studies the authors used a direct counting method by epifluorescence microscopy, thereby including non-viable cells. Bacterial concentrations reported from the Arctic, European Alps, and Tateyama Mountains (Japan) range from 1.1 × 10^4^ to 2.3 × 10^5^ cells/ml (Anesio and Laybourn-Parry, 2012), and specifically from the GrIS, 10^3^–10^6^ cells per ml of melted ice, and 10^3^–10^4^ cells per ml of supraglacial snow (Stibal et al., 2015). Our results ranged from 2 × 1 to 5 × 10^5^ of CFU per 100 ml (Figure 5). Samples with the highest abundance were cryoconite, dark ice, and supraglacial water.

### Air-Deposited Mycobiota Undergoes a Selection Process Driven by the Specific Conditions of Various Supraglacial Habitats

In contrast to the general trends in fungal community distribution in temperate zones, which shows prevalence of filamentous ascomycetous fungi, terrestrial polar and subpolar habitats are dominated by basidiomycetous yeasts (Timling et al., 2014) and ascomycetous black yeasts (Onofri et al., 1999). Comparison studies of fungal communities in cold water-ice-based environments in the Arctic are rare and mostly limited to selected habitats on Svalbard, such as cryoconite (Singh and Singh, 2012; Edwards et al., 2013) and subglacial ice (Sonjak et al., 2006; Butinar et al., 2007). We reveal here the presence of an abundant and diverse fungal population in GrIS surface ice and melt water samples, which is composed of members of Ascomycota, Basidiomycota, Chytridiomycota, and Rozellomycota phyla. Many of the fungal species identified in this study have also been found by Rämä et al. (2016) in North Atlantic driftwood indicating a surprising ability of certain fungal species to inhabit environments as different as glacial and marine. Our cultivation approach served to indicate differences in species composition between habitats, although substantial overlap of the most frequent species between some habitats was apparent, as highlighted by the PCoA of the fungal cultivable diversity (Supplementary Material). This overlap was more pronounced in our amplicon sequencing data that revealed a high similarity and low diversity in fungal taxa across all habitats except snow, and a high abundance of sequences assigned to the class *Microbotryomycetes* (Basidiomycota), as previously observed for Icelandic (Lutz et al., 2015) and Patagonian (Duo Saito et al., 2018) glaciers. We found high proportion of Chytridiomycota sequences in cryoconite samples and the presence of this phylum in all glacial samples, except snow. This is similar to other observations from Antarctic ice-covered lakes (Rojas-Jimenez et al., 2017), Svalbard cryoconites (Edwards et al., 2013) and Patagonian ice and snow (Duo Saito et al., 2018).

The majority of species from snow, determined by both cultivation and amplicon sequencing, belonged to Pleosporales, the largest order in the Dothideomycetes, comprising a quarter of all dothideomycetous species (Kirk et al., 2008). Within the Dothideomycetes, *Aureobasidium* and *Cladosporium* (represented in the samples between 0.3 and 1.2%) are two genera known for their stress tolerance, and are consistently isolated from extreme environments (Gunde-Cimerman et al., 2003; Turchetti et al., 2008; Zalar et al., 2012). Many isolates from snow belonged to various *Penicillium* spp., but *P. bialowiezense* was the only penicillium that was also recovered from dark ice. Snow and dark ice fungal communities were most dissimilar, using both cultivation and NGS techniques. The significant variation in fungal taxa between ice and snow samples suggests that the communities in ice and supraglacial water are far from being simple collections of aeolian deposits. This indicates that after deposition they have undergone a process of selection and enrichment in accordance to the specific conditions within the habitat in question.

The dominant fungi both cultivated and sequenced from dark ice were yeasts *Oleoguttula mirabilis, Rhodotorula “svalbardensis”* and *Glaciozyma antarctica*-like, (sporadically also isolated from clear ice as well). *Oleoguttula mirabilis*, reported for the first time in the Arctic, is a new species from a recently described genus of black meristematic fungi (Egidi et al., 2014), previously known only from the Antarctic rocks as members of the cryptoendolithic communities (Selbmann et al., 2005, 2008; Egidi et al., 2014). Considering its rock associated life style, in GrIS *O. mirabilis* could be associated with the mineral dust present on the dark ice and reported to play an important role as nutrient source for the ice algae (Stibal et al., 2017) and acting as hot spots of algal growth (Stibal et al., 2017). The high presence of *O. mirabilis* in dark ice samples could thus be explained by dust particles fallen with the snow and embedded within the EPS of ice algal aggregations. *Rhodotorula svalbardensis* is a novel psychrophilic yeast previously isolated only from glacier cryoconite holes of Svalbard (Singh et al., 2014). However, its existing description is not valid due to the undetermined type strain and missing name in either Mycobank or Index Fungorum. This is the first report on its occurrence on ice and cryoconite samples in the GrIS. *Glaciozyma antarctica*, formerly *Leucosporidium antarcticum* (Turchetti et al., 2011), is considered a true psychrophile, typical of cold environments, described previously from Alpine and/or Apennine glacier-associated sediments and/or Antarctic soil and sea water (Turchetti et al., 2011). It has been widely studied due to its high production of antifreeze proteins and cold-active enzymes of potential biotechnological importance (Turkiewicz et al., 2003; Ramli et al., 2012; Hashim et al., 2013). Besides these dominant species additionally many different yeasts were sporadically isolated with species belonging mainly to genera *Mrakia* and *Phenoliferia* identified in previous studies from glacial habitats, particularly glacier melt-water (Margesin and Fell, 2008; Branda et al., 2010; Pathan et al., 2010). The same melanised *Cladosporium* species as isolated from snow, representing a different fungal diversity, were also isolated from dark ice, suggesting deposition by air-borne spores.

Ascomycetous genera, such as *Acrodontium*, *Epicoccum*, *Preussia*, snow mold fungus *Microdochium nivale* (Hoshino et al., 2009), which contain species recognized as plant endophytes and plant pathogens, reported mainly from boreal, polar and alpine environments (Sati and Belwal, 2005; Sogonov et al., 2005; Bridge and Newsham, 2009), and basidiomycetous *Bjerkandera* have only been retrieved from dark ice samples. Brown et al. (2015) suggested that snow algae might act as an environmental filter that structures the snow-borne fungal community. Ice algae may perform a similar role on dark ice of GrIS. In the dark ice GrIS surface, ice algal abundances can reach up to 10^4^ cells/ml and their ability to accumulate organic carbon on the ice surface was previously demonstrated by net ecosystem production measurements (Yallop et al., 2012; Williamson et al., 2018). High primary production of the ice algae represents an important source of organic matter and nutrients (Brown et al., 2015) that may support dense fungal and bacterial communities. Brown et al. (2015) already reported a co-occurrence of snow algae with the snow-borne fungal community. Furthermore, ice algae are amongst the closest living relatives to land plants. Recent phylogenetic analyses of hundreds of proteins concluded that the Zygnematophyceae constitute the streptophyte algal group that branches closest to embryophytes (de Vries and Archibald, 2018). Ancestral streptophytes likely possessed exaptations that provided them with an advantage to terrestrial life. One of the several important adaptations required for a transition from aquatic to terrestrial habitats was the ability to form symbiotic relationships with fungal communities in order to access vital nutrient resources. Members of *Helotiales* found in this study are Ascomycetes related to rare mycorrhizal fungi, which are associated with the plant family Ericaceae growing in polar areas (Newsham et al., 2009; Tedersoo et al., 2009; Walker et al., 2011). Therefore, in addition to simply acting as decomposers, fungi could have positive or negative, not yet described, relationships with the ice algae. Cryoconite sampled with both amplicon sequencing and cultivation techniques highlighted a low fungal diversity. Sample plates from cultivation were dominated by *Articulospora* sp., a fresh water fungus belonging to the family Helotiales, that was also found when plating concentrated ice algae (data not shown). Its presence was observed in close association with clumps of dead and alive ice algae, kept together by algal EPS, suggesting a potential role in the degradation of the ice algae. *Articulospora* has been previously described in cryoconite holes in Svalbard (Singh and Singh, 2012; Edwards et al., 2013; Singh et al., 2016) and its role as an important carbon decomposer was hypothesized. This result was confirmed by amplicon sequencing (*Cladochasiella divergens*; 0.11–0.15%) that furthermore showed the presence of the plant-associated black yeast genus *Cladophialophora* (9.2–12.6%). Other species obtained by cultivation were mainly basidiomycetous yeasts, such as *Mrakia* sp., *Rh. “svalbardensis”*, and *Glaciozyma antarctica*-like typical for polar habitats (see above).

Finally, we observed a substantial difference in cultivable fungal diversity between the sampling seasons of 2016 and 2017. The majority of isolates cultivated from 2016 sampling belonged to *Penicillium bialowiezense*-like (data not shown), dominating in all the environments, while in 2017 the isolates were much more diverse and belonged to 33 different genera. Two possible reasons for the observed differences could be the different snow conditions on the GrIS between 2016 (after the snow melting) and 2017 (before the snow melting), and the use of a wider selection of media in 2017. The results of amplicon sequencing, however, did not show such stark dissimilarity between the seasons, possibly pointing to insufficient sampling of the total fungal community with cultivation methods or their greater bias.

### Bacterial Communities

Heterotrophic bacteria are common within the various supraglacial habitats where they play an important role in nutrient cycling of decaying organic matter and remineralization of the limited nutrients available. Bacterial populations of the GrIS supraglacial environments have been studied for years (Yallop et al., 2012; Cameron et al., 2015a,b; Musilova et al., 2015; Stibal et al., 2015; Williamson et al., 2018), facilitating a comparison of our 16S rRNA results with previous studies. However, none of these studies have targeted the bacterial community associated with the ice algae in dark ice on the SW margin of the GrIS.

In line with previous studies (Larose et al., 2010; Edwards et al., 2011; Cameron et al., 2012) Proteobacteria (mainly alpha-proteobacteria), Bacteroidetes and Actinobacteria were present in all our samples. This result was supported by amplicon sequencing and by cultivation. Dark ice 2017 was dominated in particular by the phylum Armatimonadetes (order FW68) that was present the same year to a lesser extent also in supraglacial water and clear ice. Classes of this phylum have been recovered from a broad range of temperatures and environmental niches, such as terrestrial and aquatic habitats, human skin, anaerobic bioreactors and waste water treatment plans, geothermal soils and springs (Lee et al., 2014). Armatimonadetes strains are commonly detected in areas dominated by photosynthetic bacteria or eukaryotes and they are most probably involved in the degradation of photosynthetic plant and microbial biomass and polysaccharide-based substances (Burns et al., 2004; Ley et al., 2006; Isenbarger et al., 2008; Pope and Patel, 2008; Shao et al., 2011; Lee et al., 2014). Class Ktedonobacteria (Chloroflexi) was especially abundant in cryoconites, but also present in all other samples except snow. Ktedonobacteria are Gram-positive and aerobic, with a complex morphology, forming branched mycelia with spores similar to mycelia-forming actinomycetes. They are prominent in extreme environments such as volcanic, Antarctic, and cave ecosystems (Gomez-Alvarez et al., 2007; Barton et al., 2014; Kim et al., 2015; Tebo et al., 2015). Snow samples were substantially different from all other sampled habitats and, as reported previously (Amato et al., 2007; Cheng and Foght, 2007), were dominated by Actinobacteria, usually identified as key components in cryoconite (Gokul et al., 2016).

## Conclusion

This is the first report of fungal communities in GrIS water-based ecosystems and one of the few culture independent studies of microbial diversity in ice. In our amplicon sequencing based study of the microbial variety of GrIS supraglacial habitats, we described more diverse bacterial communities than fungal communities. Combining different approaches was thus beneficial in these extreme environments with low biodiversity. Dark ice fungal communities were characterized by plant pathogenic fungi and endophytes and a much higher fungal and bacterial abundance than in other supraglacial habitats on the GrIS, underlining its distinctiveness and suggesting that ice algal clumps probably influence the associated microbial diversity and abundance. In particular, fungal roles in the prospering and degradation of ice algal blooms should be investigated further. Since the darkening of ice (in part due to algal blooms) has the potential to significantly increase GrIS surface melt, interactions between all microbial communities in this specific habitat should be studied in more detail.

## Data Availability

The datasets generated for this study can be found in GenBank, MK460310–MK460422, MK454775–MK454866, MK453054–MK453127, PRJNA507743, and PRJNA517071.

## Author Contributions

LP collected the samples with the support of AA and CW. LP performed the wetlab analyses. CG performed the bioinformatic analyses of NGS data. LP and CG analyzed the data. LP, NG-C, and CG interpreted the data with contributions from AA and CW. LP drafted the manuscript. All authors contributed to design of the experiments, revised the manuscript draft, and approved the final version of the manuscript.

## Conflict of Interest Statement

The authors declare that the research was conducted in the absence of any commercial or financial relationships that could be construed as a potential conflict of interest.
